# Antimicrobial and antibiotic-potentiating effect of calcium peroxide nanoparticles on oral bacterial biofilms

**DOI:** 10.1038/s41522-024-00569-7

**Published:** 2024-10-15

**Authors:** Neha Bankar, Lorenz Latta, Brigitta Loretz, Bashar Reda, Johanna Dudek, Hendrik Hähl, Matthias Hannig, Claus-Michael Lehr

**Affiliations:** 1 Helmholtz Institute for Pharmaceutical Research Saarland (HIPS), Helmholtz Centre for Infection Research (HZI), Saarland University, 66123 Saarbrücken, Germany; 2 Department of Pharmacy, Saarland University, 66123 Saarbrücken, Germany; 3 Clinic of Operative Dentistry, Periodontology and Preventive Dentistry, University Hospital, Saarland University, Building 73, 66421 Homburg/Saar, Germany; 4 Experimental Physics and Center for Biophysics, Saarland University, 66123 Saarbrücken, Germany; 5 Present Address: Department of Periodontology, School of Dentistry, Al- Shahbaa Private University, 66123 Aleppo, Syria

**Keywords:** Antimicrobials, Dental conditions

## Abstract

Bacterial biofilms represent a prominent biological barrier against physical and chemical attacks. Disturbing the anaerobic microenvironment within biofilms by co-delivery of oxygen appears as a promising strategy to enhance the activity of an antibiotic. Here, we report the effect of oxygen-producing calcium peroxide nanoparticles (CaO_2_ NP) in combination with tobramycin sulfate (Tob). On *Pseudomonas aeruginosa* PAO1 biofilms in vitro, the additive effect of CaO_2_ NP towards Tob activity enhanced biofilm eradication by 2 log compared to Tob alone. For natural biofilms grown in the oral cavity of human volunteers in situ, treatment by CaO_2_ NP alone slightly increased the fraction of dead bacteria from 44% in various controls, including Tob alone, to 57%. However, the combination of CaO_2_ NP with Tob further increased the fraction of dead bacteria to 69%. These data confirm the intrinsic antimicrobial and antibiotic-potentiating effect of CaO_2_ NP also in a clinically relevant setting.

## Introduction

Bacteria can adapt to hostile environment by the formation of biofilms^1^. A biofilm matrix is constructed by the bacteria to protect themselves from chemical and physical harm^2^. Biofilm complexity varies due to the presence of extracellular polymeric substances (EPS), extracellular DNA, proteins, lipids, and other components which help the bacteria survive longer in harsher conditions^2,3^. The hydrogel structure of bacterial biofilms contributes as a penetration barrier against human immune defense, disinfectants, and antimicrobial agents^4^. As a biofilm, bacteria can often tolerate much higher antibiotic treatments than when treated after dispersal in to their planktonic form^5^. In bacterial infections the mode of growth can transit between planktonic and biofilm form, contributing to the difficulties to eliminate some bacterial infections with antibiotics. Pathogens such as *Acinetobacter baumannii*, *Pseudomonas aeruginosa*, and *Enterobacteriaceae* are critically prioritized due to their ability to cause fatal infections in humans^6^.

In bacterial biofilms, an important limiting factor that leads to hostility towards treatment is the nutrient and oxygen gradient^4,7^. Regions with hypoxia in the biofilm causes a metabolic shift in the survival of pathogens resulting in anaerobic respiration^8^. Such bacteria become more tolerant towards antibiotic treatment and may develop antimicrobial resistance^4,7^. Lack of oxygen has also been reported to favor the development of solid tumors^9^ which may perhaps be overcome by treatment with reactive oxygen species (ROS)^10^. In this context, calcium peroxide has already been extensively studied^11,12^. When solid peroxides like calcium peroxide come in contact with water, the intermediate results in the generation of hydrogen peroxide which is a ROS, and subsequently releases oxygen^13^. This has led to the idea of using calcium peroxide also in antibacterial therapy as a bacteriostatic reagent^14^. The peroxide released causes bacterial death and helps in wound healing and bone regeneration^15,16^. It was also studied in the treatment of individual bacterial species like *Porphyromonas gingivalis* causing periodontitis^17^. Hydrogen peroxide is also a major content in commercially available dental products, such as gels and pastes^18,19^. Especially calcium peroxide is used in tooth whitening and bleaching products^20,21^. According to the scientific committee on consumer products of the European Commission, safety for peroxide containing products was analyzed and the findings suggested that when calcium peroxide was used at 0.5% in toothpaste it may be considered as safe to use more than once a day with no adverse reactions^22^. Studies indicating the bacteriostatic effect of calcium peroxide^14,15^, and its plausible effect on teeth^20,21^; suggested the possibility of repurposing calcium peroxide for the treatment of oral infectious diseases.

The Center for Disease Control and Prevention had reported a serious threat against multidrug-resistant *Pseudomonas aeruginosa* in 2019^23^. Such infections are usually treated with the broad-spectrum aminoglycoside antibiotic tobramycin at a dose of 1 mg/kg of body weight injected intramuscularly^24^. According to the list for medically important antimicrobials in human medicine released by the WHO in 2024, aminoglycosides are prioritized as critically important antimicrobials in cases of a very high risk of antimicrobial resistance^25^. Exposure to conditions like a low nutrient, hypoxia, and competition in multi-bacterial infections causes bacterial stress and fosters biofilm formation. In particular, *P. aeruginosa* biofilm formation due to stress conditions is known^26,27^ as well as its biofilm-dependent tolerance against tobramycin^28^. Therefore, *P. aeruginosa* in vitro biofilm was selected to investigate the effects of combination treatment with calcium peroxide and tobramycin^14,28^. In addition, we aimed for a native multi-species biofilm^29,30^. The importance of the oral microbiome on human health^31,32^, the biocompatibility of calcium peroxide^22^ and the availability of human native samples suggested the use of oral in situ formed biofilm for our studies^33,34^.

In this study, we prepared the calcium peroxide nanoparticles for the generation of oxygen and ROS in the biofilms. We investigated the combination of Tobramycin sulfate (Tob) with Calcium peroxide nanoparticles (CaO_2_ NP) on in vitro *P. aeruginosa* PAO1 biofilms. The dose dependency to eradicate the infection, due to the medical importance, was also studied. Calcium may be hypothesized to also contribute to improving biofilm permeability. To decipher the anti-biofilm effect of oxygen from calcium supply by CaO_2_ NP, we included calcium chloride (CaCl_2_) for comparison. Effects on bacterial gene expression were quantified by PCR for selected marker genes. Finally, the CaO_2_ NP and Tob co-treatment was evaluated on natural biofilms grown in the oral cavity of human volunteers in situ.

## Methods

### Materials

Calcium chloride was obtained from BDH^®^ prolabo VWR chemicals (Darmstadt, Germany), ammonia solution, 25% was obtained from Suprapur^®^, Supelco (Merck KGaA, Darmstadt Germany), hydrogen peroxide, 35 wt % solution in water Acros Organics™, fetal calf serum (FCS), trypsin, DMEM (low glucose, GlutaMAX™ Supplement, pyruvate) (Gibco™) cell culture medium, RNAProtect^®^ bacteria reagent Qiagen, SYBR green real-time qPCR master mix (SYBR MM) were all purchased from Thermo Fisher Scientific (Darmstadt, Germany), tobramycin sulfate (salt), PEG 200 (P3015), glutaraldehyde 25% solution in water, 3-[4,5-dimethylthiazol-2-yl]-2,5 diphenyl tetrazolium bromide (MTT), paraformaldehyde, luria bertani (LB) broth, hexamethyldisilazane (HMDS), PBS (Dulbecco’s Phosphate buffer saline), NaOH pellets, ethanol (99.95% *v/v*) were all purchased from Sigma-Aldrich (Merck KGaA Darmstadt, Germany), BreathSeal foils were purchased from Greiner Bio-One (Frickenhausen, Germany), paraformaldehyde 16% aqueous solution EM Grade (Electron Microscopy Sciences), qScriber™ cDNA synthesis kit from highQu, RNeasy^®^ Micro Kit from Qiagen, TURBO™ DNase kit, Live/Dead^®^ BacLight™ Bacterial Viability kit L7012 and L7007 were all purchased from Invitrogen, Thermo Fisher Scientific (Darmstadt, Germany). Poly-lactic-co-glycolic-acid (Resomer RG 503 H) was purchased from Evonik Industries (Darmstadt, Germany), the average particle size as determined by dynamic light scattering (DLS), Zetasizer Nano-ZS (Malvern Instruments, Worcestershire, U.K.) was 231 ± 46 nm, PDI < 0.3.

Gingival fibroblast cells (human gingival fibroblast cells) were obtained as immortalized cells from patients. *P. aeruginosa* PAO1 (DSM No. 22644, DSMZ, Braunschweig, Germany) was used.

### Preparation and characterization of CaO_2_ NP

Calcium peroxide nanoparticles were prepared by nanoprecipitation^12,35,36^. PEG 200 (16 mL) and ammonia solution (2 mL) were added to a stirred solution of calcium chloride (400 mg) in distilled water (2 mL); this mixture was heated at 70 °C. Under continuous heating and stirring conditions (750 rpm), addition of H_2_O_2_ (2 mL) at a rate of 50 µL/min was performed. To precipitate the nanoparticles, pre-heated (at 50 °C) NaOH solution (0.1 M) was added till the mixture pH was 13. The precipitate was immediately centrifuged at 5000 rpm for 5 min and supernatant was discarded. To remove excess PEG200 residue, precipitate was washed three times with 10 mL NaOH solution: distilled water (1:1); centrifuged each time at 5000 rpm for 2 min. The product was dried in vacuum oven at 80 °C at 0 mbar for 3 h.

For particle size analysis of calcium peroxide nanoparticles Horiba LA-950V2 Laser Scattering Particle Size Distribution Analyzer (Retsch GmbH, Haan, Germany) was used. CaO_2_ NP powder was dispersed in ethanol (99.95% v/v) using an ultrasonic probe Sonicator S-250D model (Branson Ultrasonics, USA) at 10% intensity for 20 s and the particle size was measured at room temperature. The size distribution and morphology of CaO_2_ NP were confirmed with scanning electron microscopy. CaO_2_ NP dry powder and CaO_2_ NP dispersed in ethanol were placed onto stubs using double-sided carbon tape. After complete drying of sample, gold sputtering was performed in QUORUM Q150R ES, (Pfungstadt, Germany) pumped coater under vacuum. Images were obtained using EVO HD15 microscope (Zeiss, Oberkochen, Germany).

Oxygen concentration of water can be determined using a dissolved oxygen meter^12,37^. When the CaO_2_ NP were added to deoxygenated water, due to the reaction with water (Eq. 1); oxygen was released into the surrounding^38^.

1 $$2\left(\right.{\rm{CaO}}_{2}+4{{\rm{H}}}_{2}{\rm{O}}\to 2\left({\rm{Ca}}{\left({{\rm{OH}}}\right)}_{2}\right)+2({\rm{H}}_{2}{{\rm{O}}}_{2})\to 2\left({\rm{Ca}}{\left({\rm{OH}}\right)}_{2}\right)+2{\rm{H}}_{2}{\rm{O}}+{\rm{O}}_{2}\uparrow$$

To determine the dissolved oxygen content of water a dissolved oxygen meter (sensor, InLab OptiOx) (Mettler-Toledo AG, Analytical, Schwerzenbach, Switzerland) was used. To determine the oxygen release 20 mg samples were placed in a 2-necked round bottom flask. Deoxygenated distilled water (pre-bubbled with nitrogen gas) with oxygen concentration of <1 mg/L was added to these samples. Samples were stirred at 32 rpm and 150 m bar vacuum using R-300 Rotavapor (Büchi, Flawil, Switzerland). The oxygen concentration of water was checked after 20, 60, 120, 180, 240 min for each sample. The oxygen release from CaO_2_ NP, PLGA particles, and O_2_-PFH liposomes were compared.

To determine the H_2_O_2_ release from CaO_2_ NP, Pierce™ quantitative peroxide assay kit was used, calibration curves in triplicates for H_2_O_2_ concentration from 7.5 to 90 µM/mL were plotted and four different batches of CaO_2_ NP were incubated with the reagent for 25 min and absorbance at 595 nm was measured.

To determine the effect of CaO_2_ NP on gingival fibroblast cells, MTT assay was performed^39^. 10,000 cells per well were cultured at 37 °C in a 5% CO_2_ incubator in DMEM, low glucose, GlutaMAX™ Supplement, pyruvate medium containing 10% FCS for 2 days in a 96 well plate. 100 µL samples with increasing concentration of CaO_2_ NP (1, 8, 16, 32, 64, 96, 128, 1000 µg/mL) and 100 µg/mL O_2_- PFH liposomes were placed on cells in HBSS buffer, after 4 h cells were washed and treated with MTT reagent (450 µg/mL) for 3 h. DMSO was added to each well and absorbance at 550 nm was recorded. Analysis of controls with 1% Triton-X in HBSS for complete cell death as positive control, and only HBSS buffer for 100% viability of cells as negative control were also performed (as in Eq. 2).

2 $$\% {cell\; viability}=\frac{{A}_{{sample}}-{A}_{{positive\; control}}}{{A}_{{negative\; control}}-{A}_{{positive\; control}}}* 100$$

### In vitro studies on PAO1 biofilms

PAO1 was initially inoculated in LB medium for 16 h at 37 °C, 180 rpm in a shaking incubator. On the next day, bacterial suspension was centrifuged at 5000 rpm for 10 min at 4 °C and re-suspended in PBS. Sterile M63 medium was prepared by modifying the carbon source to arginine^40^. To cultivate PAO1 biofilms, M63 medium was diluted with the obtained bacterial suspension to get an OD of 0.01, corresponding to the CFU of approximately 1*10^7^/mL of bacteria. This was incubated in 96 well plates covered with BreathSeal foil at 37 °C for 48 and 72 h without shaking to get differently old biofilms of PAO1. To quantify the effect of CaO_2_ NP and CaCl_2_ on the 48 h and 72 h PAO1 biofilms, were treated with 16 and 32 µg/mL concentration, resp., for 2 h. Further, study the effect of Tob in combination or alone, Tob was used at increasing concentrations of 64, 128, 256 µg/mL, resp. Biofilms were treated overnight with the above-mentioned scheme analyzed using CFU counting assay for viability studies. Controls were also tested on 72 h PAO1 biofilms, firstly they were treated with 36 and 300 µg/mL blank liposomes, PFH liposomes, and O_2_-PFH liposomes alone and in combination with 256 µg/mL Tob overnight and analyzed with CFU assay. Concentration of 36 µg/mL liposomes was selected as a comparison to CaO_2_ NP and 300 µg/mL was selected based on in vitro experiments conducted by Hu et al.^41^. Secondly, colistin was tested at 8, 16 and 32 µg/mL concentrations in combination to 32 µg/mL CaO_2_ NP and CaCl_2_ in same earlier mentioned protocol.

### Microscopical and morphological analysis of PAO1 biofilm

Samples were analyzed using fluorescence microscopy Leica DMi8 Confocal laser scanning microscope (Leica, Mannheim, Germany). The live/dead staining was performed using BacLight™ Live/Dead® Staining Kit L7007. Samples were treated in 16well microscopic slides. Treatment with staining solution Component A, was done differently considering the biomass present for treated samples; i.e. for higher biofilm content samples observed (Untreated Control, CaO_2_ NP treated and, CaCl_2_ treated) 2.5 µL stain was used. However, for samples with less biofilm mass 1.5 µL staining solution was added. After addition of staining solution the slide was kept in dark for 15 min at room temperature. Samples were then observed under the microscope using a 10x objective (HC PL Fluotar 10X/0.3 Dry, Leica, Germany) and analyzed with LASX software from Leica Application Suite X. Morphological analysis was also performed using SEM microscopical analysis. For the ease of imaging PAO1 biofilms were grown on sterilized hermatic sealing lids (used in DSC (Differential Scanning Calorimetry) analysis) placed in 24 well plate. Samples were fixed using 4% PFA solution in PBS for 2 h at room temperature. These samples were then treated with 30–100% ethanol dilution series for 10 min at each concentration. A final treatment with HMDS for 10 min was performed to completely dehydrate the biofilms and dried overnight at room temperature. The lids containing dried biofilms were carefully placed on SEM sample holder stubs with a double-sided carbon tape. Gold sputtering at argon atmosphere vacuum was performed in QUORUM Q150R ES, (Pfungstadt, Germany). SEM images were obtained using EVO HD15 microscope (Zeiss, Oberkochen, Germany).

### Preparation of O_2_-PFH liposomes

For the control experiments performed on the PAO1 biofilm, O_2_-PFH liposomes were formulated^41^. DOTAP:DOPE:HSPC:DSPE-PEG2000:Chol in a molar ratio of 10:20:40:10:20 to make a total of 30 mg content were dissolved in 10 mL chloroform. The thin film hydration method for liposome formation was used. For chloroform evaporation the mixture was rotated at 100 rpm and 390 m bar vacuum at 37 °C using R-300 Rotavapor (Büchi, Flawil, Switzerland). For the formation of liposomes 10 mL MilliQ water was added and mixture was sonicated in a bath sonicator for 10 min with shaking. To formulate the PFH liposomes, 1.2 mL PFH was added to the liposomes and this was sonicated with ultrasonic probe Sonicator S-250D model (Branson Ultrasonics, USA) at 10% intensity for 15 min on an ice bath. To load oxygen to PFH liposomes air was bubbled for several minutes and oxygen concentration was checked before using it in experiments. The particle size as determined by Zetasizer Nano-ZS (Malvern Instruments, Worcestershire, U.K.) was 262 ± 40 nm, PDI < 0.1.

### Expression analysis of bacterial genes in response to treatments

Gene expression for several genes in *P. aeruginosa* were selected based on the processes needed for biofilm virulence and survival. The following genes were selected as; hcnA operon is a subunit of the Hydrogen Cyanide Synthase (HCN) responsible in anaerobic respiration and virulence generation during multi-bacterial infections, the gene expression upregulates for hcnA during micro-aerobic conditions^42^. Pyocyanin is a phenazine produced by *P. aeruginosa* as a virulence factor to establish its niche, responsible for depletion of antioxidants and increasing antibiotic resistance; encoded by the phzA operon^43^.The quorum sensing involved for cell to cell communication in *P. aeruginosa* biofilm, and in regulation of cell density involves the expression of 4-hydroxy-2-alkylquinolines (HAQs) by the pqsA operons^44^. Similarly, mvfR is involved in *P. aeruginosa* quinolone signaling (PQS), a quorum sensing path responsible in synthesis of HAQs and the generation of several compounds like elastases, pyocyanin in the biofilm responsible for virulence generation^45^. In the *P. aeruginosa* quorum sensing involving the homoserine lactones rhlR is responsible for the regulation of virulence related to the rhamnolipid generation^46^. The sigma factors rpoS and rpoD are involved in *P. aeruginosa* quorum sensing at different phases of growth cycle, the earlier involved during stationary phase extracellular virulence factors generation, while the latter is involved in transcription of several housekeeping genes^47^. The efflux pump protein mexG encoded by mexGHI-opmD operon responsible for antimicrobial resistance was also studied for its expression^48^. Under carbon limiting conditions, the extracellular respiration with Nicotinamide Adenine Dinucleotide hydrogen is governed with the nadB operons^49^. In the biofilm formation, algD is one of the genes required in the production of alginate^50^. gyrA is the DNA gyrase genome replication housekeeping gene, it is considered stable during treatments so is used as the reference gene in the study^51^. After the selection of the above genes, the primer sets for the above-mentioned genes were acquired (Supplementary Table 1)^52^.

To isolate RNA from treated samples (total volume 400 µL, 48 well plate); equivalent amount of RNAProtect^®^ bacteria reagent was added to samples and vortexed for 10 min at room temperature. This mixture was then centrifuged for 10 min at 5000 *g* at 4 °C, supernatant was discarded and the pellet was freezed at −80 °C for 2 h before using it in RNA isolation and purification. RNA was isolated by adding 96 µL of Tris-EDTA (20 mg/mL) buffer and 4 µL lysozyme to the pellet, vortexed for 15 min; to this mixture 350 µL of RLT buffer supplemented with 1% ß-mercaptoethanol was added and incubated at −70 °C for 1 h. After thawing the sample it was further loaded on QIAshredder column and centrifuged at 12,000 *g* for 2 min at 4 °C. After centrifugation the flow through containing RNA was isolated and mixed with equivalent amounts of ethanol. The RNA was purified following RNeasy^®^ Micro Kit instructions including on-column DNA digestion. For elution of RNA 28 µL RNAse free water was used, followed by an additional step of DNAse digestion using 1 µL TURBO™ DNase reagent for 1 h, at room temperature. Finally, the concentration of purified RNA was determined using NanoQuant Plate™ in a plate reader (CytoSparc Tecan Instruments, Männedorf, Switzerland). cDNA was synthesized from the isolated RNA using qScriber™ cDNA synthesis kit protocol, in the Applied Biosystems™ Thermal Cycler (Thermo Fisher Scientific, Darmstadt Germany), applying 150–300 ng RNA per 20 µL cDNA reaction and incubation at 45 °C for 30 min. After the reaction, enzymes were deactivated at 85 °C for 10 min and the cDNA was stored at −20 °C. Before using the synthesized cDNA it was further diluted (to make 50 µL) and used for qPCR analysis. qPCR was performed using the Bio Molecular Systems Mic qPCR Cycler (Oldendorf, Germany). For this analysis primer sets for selected genes, hcnA, pqsA, phzA, rpoS, mexG, rpoD, rhlR, mvfR, nadB, algD, and gyrA were mixed with SYBR Mastermix and cycle number (C_q_ values) were determined using Bio-Rad CFX Manager analysis software. gyrA was used as the control gene for the regulation calculations. As residual DNA contamination could not be avoided entirely, a –RT control was conducted for each reaction in parallel. To normalize the samples at least 10 cycle difference between the +RT and –RT values was considered, and such values of expression for samples were further calculated using the ΔΔCq method in comparison to the reference gene. The expression fold change was calculated and plotted. The experiments were performed thrice with at least three replicates in total while calculating the fold change.

### In situ oral biofilm treatment protocol

The oral biofilm was collected from two volunteers. For in situ biofilm formation enamel specimens were prepared from bovine incisor teeth. Enamel slabs (ca. 3 × 4 × 1.5 mm) were polished by wet grinding with a final grain size of 2500 (Buehler, Düsseldorf, Germany) and purified before oral exposure as described previously^53^. Then the specimens were mounted on individual splints (Duran, SCHEU-DENTAL GmbH, Iserlohn, Germany) using silicone impression material (PRESIDENT light body, Coltène/Whaledent GmbH + Co. KG, Langenau, Germany) and exposed intraorally for 48 h (Fig. 7a). During oral exposure, specimens were not subjected to any cleaning procedures; furthermore, volunteers refrained from using any agents (e.g., toothpastes or mouth rinses) for cleaning measures. During meals, splints were removed and stored in a wet chamber. Subsequently, the biofilms were treated ex situ for 18 h at 37 °C with the testing solutions. For this, the enamel specimens were washed with sterile water and individually placed in wells of a 96 well plate. For CaO_2_ NP application the nanoparticles dispersed in ethanol (20 µL) were added. After ethanol evaporation, PBS was added and gently stirred to redisperse CaO_2_ NP. Initial experiments (N*n = 1*2) involved treatment with different concentrations of CaO_2_ NP (32, 96, 144 and 192 µg/mL) and Tob (dissolved in PBS at 1000, 500, 250 and 128 µg/mL). Afterwards experiments (N*n = 5*2) with selected concentration of 96 µg/mL CaO_2_ NP and 128 µg/mL Tob or mixture of both were performed. As controls (N*n = 3*2) 96 µg/mL CaCl_2_ dissolved in PBS, 300 µg/mL O_2_-PFH liposomes and PBS (Untreated Control) were applied. Viability assessment by fluorescence microscopy was performed as previously described^54^. Briefly, specimens were stained with a SYTO 9 (all bacterial cells) and propidium iodide (dead bacterial cells) containing solution for 10 min in the dark (LIVE/DEAD® BacLight™ Bacterial Viability kit L7012). Imaging was performed using a fluorescence microscope (Axio Imager 2 Microscope, Zeiss MicroImaging, Göttingen, Germany). At least four representative randomized images per specimen were taken at a magnification of 1000-fold using the image processing software AxioVision 4.8 (Carl Zeiss Microimaging, Göttingen, Germany). The biofilm viability was estimated by counting living bacterial cells (green) and dead bacterial cells (red) using a custom-made analysis routine written in MATLAB utilizing its Image Processing Toolbox (MATLAB and Image Processing Toolbox version: 9.10.0 (R2021a) Natick, Massachusetts: The MathWorks Inc.; 2021). The routine identifies bright objects whose diameter lies within a specified range using top-hat-filtering, morphological opening as well as a watershed-segmentation to separate bacterial clusters. Objects appearing only in the red channel or in the green and in the red channel are counted as dead, those appearing only in the green channel as living bacteria.

### Statistical analysis

Unless stated otherwise, experiments were performed three times (*N* = 3), with three biological replicates (*n* = 3). Statistical significance was checked using one-way ANOVA with Tukey’s multiple comparison test using the GraphPad Prism software. In the graphical representation, error bars indicate the standard deviation in individual treatment. Statistical significance between groups was defined by *p < 0.05, **p < 0.01, ***p < 0.001, ****p < 0.0001.

## Results

### Preparation and characterization of CaO_2_ NP

**Fig. 1:**
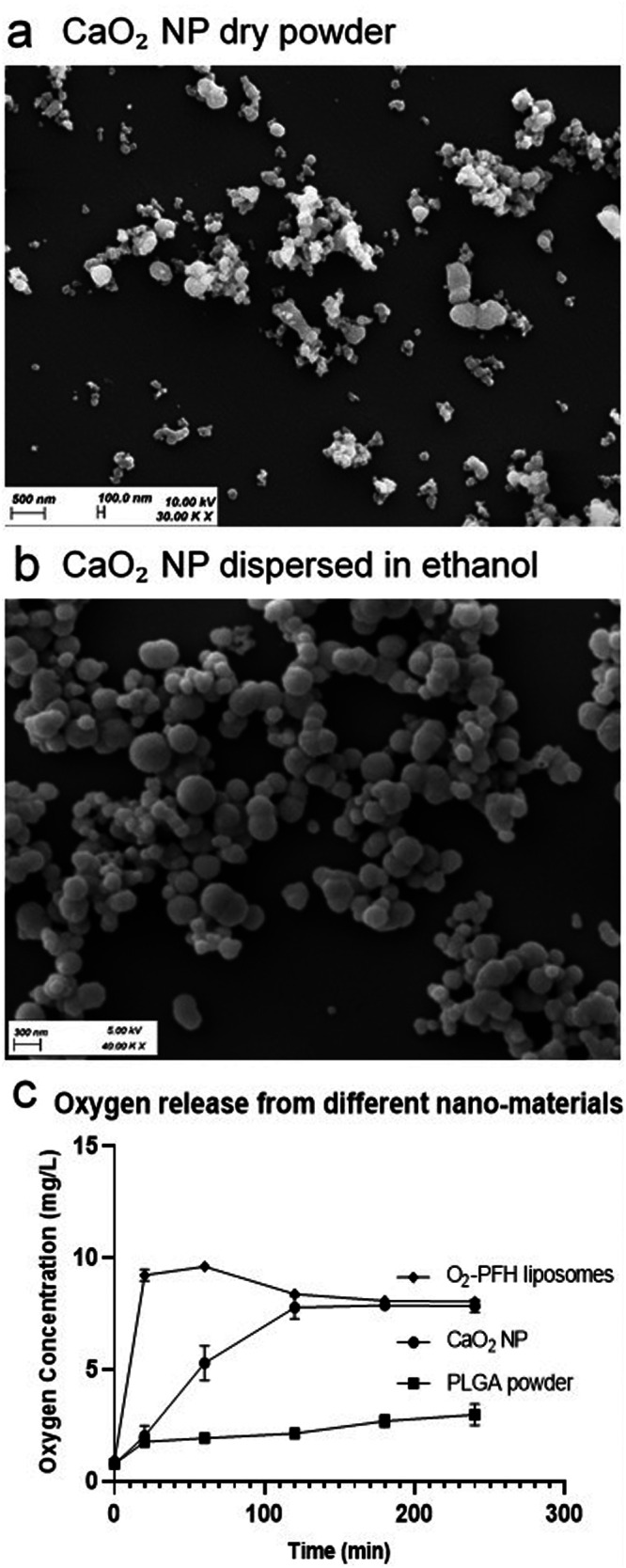
Characterization of CaO_2_ NP; SEM size and morphological analysis. (**a**) CaO_2_ NP dry powder showing spherical particle aggregation. (**b**) CaO_2_ NP dispersed in ethanol, showing smaller particles of spherical aggregation. (**c**) Oxygen release studies from different nano-materials- CaO_2_ NP increased the oxygen concentration of deoxygenated water to 7.8 mg/L in 120 min, O_2_-PFH liposomes increased the oxygen concentration of deoxygenated water to 9 mg/L in 20 min before a decrease and plateauing at 8 mg/L, PLGA particles (a non-oxygen releasing control) the oxygen concentration slowly increased but remained below 2 mg/L.

CaO_2_ NP were prepared by nanoprecipitation adapted from Sheng et al. and Rastinfard et al.^12,35^. The process required heating the reaction mixture at a temperature of 70 °C, for decreasing the degree of hydration while particle precipitation. During precipitation of particles a lower molarity (0.1 M) NaOH was used, as too fast precipitation resulted in excess agglomeration of the particles. The particle size was distributed with a median (D50) of around 120 nm and 90% diameter (D90) of around 160 nm (Table 1). SEM (Scanning Electron Microscopy) images were taken to visualize particle morphology and as a second method for particle size determination. Micrographs of the dry powder showed larger aggregates (Fig. 1a). Micrographs from the ethanol dispersion of the powder depicted segregated spherical aggregates of smaller particles (Fig. 1b). The hydrogen peroxide release after 25 min from CaO_2_ NP was 49 ± 20 µM/mL (Supplementary Fig. 1b).

**Table 1:** Size distribution of CaO_2_ NP

Particle size distribution (nm)			
Batch	D10	D50^a^	D90
1	75	112	168
2	78	122	191
3	72	106	160

^a^D50: median diameter, (*N* = 3).

Oxygen release from CaO_2_ NP was measured in deoxygenized water using a dissolved oxygen (DO) meter, reaching a maximum of 7.8 mg/L after 120 min (Fig. 1c). The observed plateau could be the result of a complete CaO_2_ NP powder reaction with water, or that the maximal dissolving capacity of water for oxygen was reached. For comparison, two other nano-materials were tested; a non-oxygen containing poly-(lactic-co-glycolic)-acid (PLGA) in the form of sub-micron size particles (231 ± 46 nm, PDI (Polydispersity index) < 0.3), and oxygen-perfluorohexane (O_2_-PFH) liposomes^41^. The DO release from PLGA remained below 2 mg/L with a very slow increase over the measurement time of 4 h, which can be considered as background of the experimental set-up. In contrast, the O_2_-PFH liposomes provided a rather steep increase of the DO concentration; within 20 min it reached nearly 9 mg/L before a small decrease and plateauing at 8 mg/L.

As a rough estimate of biocompatibility, CaO_2_ NP were used in a MTT assay determining cell viability of gingival fibroblast cells. As expected, concentration dependent cytotoxicity was observed with a lethal concentration 50% of 16 µg/mL (Supplementary Fig. 1a). The concentration of CaO_2_ NP for the in vitro biofilm studies (see below) was 16 and 32 µg/mL, resp.

### In vitro studies of CaO_2_ NP on PAO1 biofilms

**Fig. 2:**
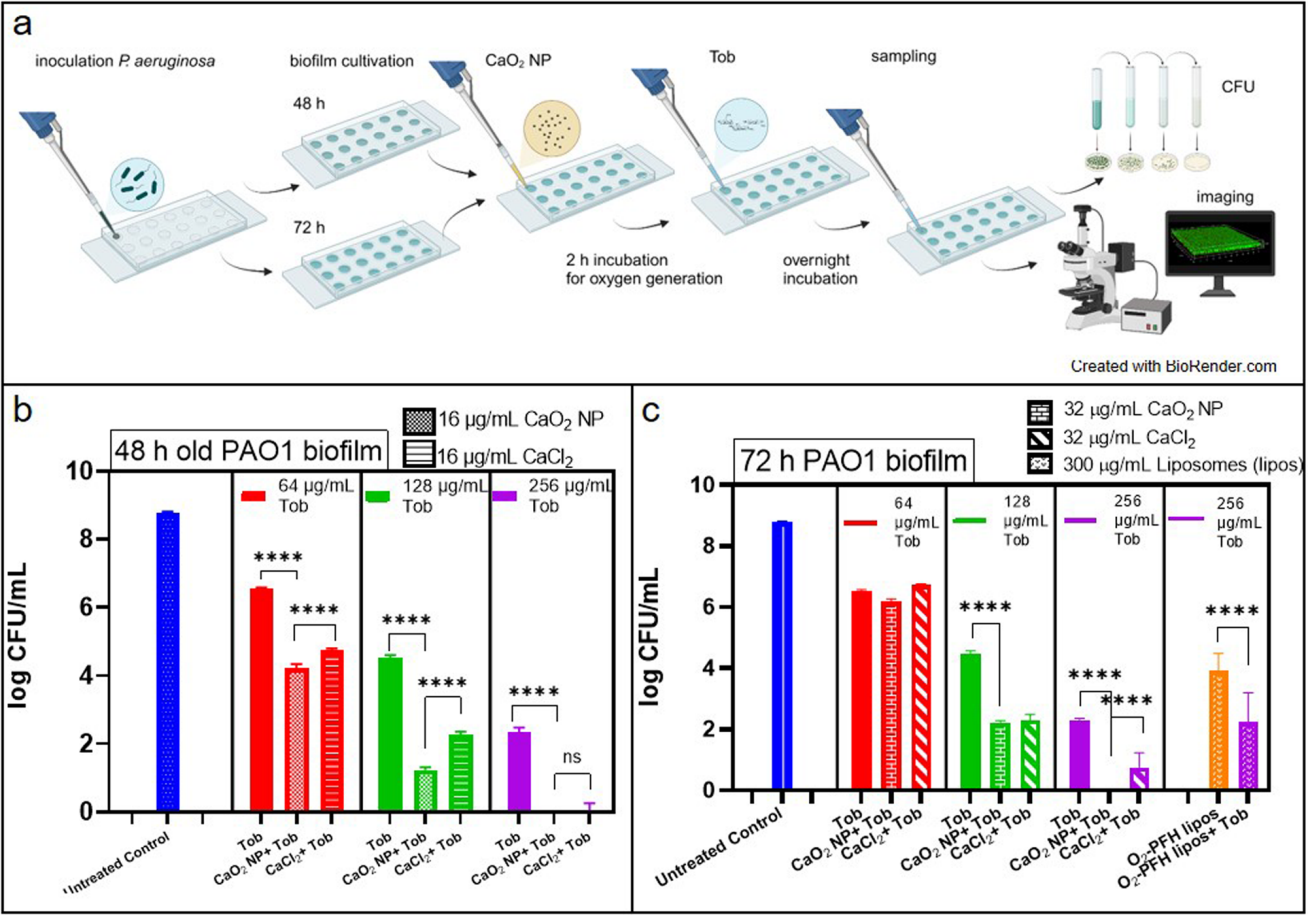
PAO1 biofilm eradication assay. (**a**) Schematic representation for treatment Scheme, 48/ 72 h old PAO1 biofilm pretreatment with oxygen generating CaO_2_ NP, with subsequent treatment of Tob overnight, analyzed by CFU assay and Confocal laser scanning microscopic image analysis, created with BioRender.com. (**b**) CFU assay for in vitro 48 h old PAO1 biofilm- Comparison between 16 µg/mL CaO_2_ NP containing both Ca^++^ and oxygen, 16 µg/mL CaCl_2_ containing only Ca^++^, with antibiotic Tob at different concentrations of 64, 128, and 256 µg/mL respectively. Biofilm eradication was complete for both 16 µg/mL CaO_2_ NP/ CaCl_2_ in combination with 256 µg/mL Tob. (**c**) CFU assay for in vitro 72 h old PAO1 biofilm- Comparison between 32 µg/mL CaO_2_ NP/ CaCl_2_ both in combination with antibiotic Tob at different concentrations 64, 128, and 256 µg/mL respectively. Biofilm eradication was complete in combination treatment with CaO_2_ NP and Tob, but not in CaCl_2_ and Tob. For comparison 300 µg/mL O_2_-PFH liposomes were tested alone or in combination with 256 µg/mL Tob. O_2_-PFH liposomes were not effective either alone or in combination with Tob in biofilm treatment. Experimental analysis performed using One-Way ANOVA with Tukey’s post hoc test. N*n = 3*3, ****p < 0.0001.

Minimum biofilm eradication concentration (MBEC) assays were performed to know the required concentration of CaO_2_ NP and Tob in combination to eradicate PAO1 biofilm. Biofilms were first grown for either 48 or 72 h and then treated with 16 and 32 µg/mL CaO_2_ NP or CaCl_2_, respectively, for 2 h, followed by overnight treatment with Tob at 64, 128 and 256 µg/mL concentration (Fig. 2a). The different time periods of biofilm growth before treatment were chosen to address the effect of biofilm age on the treatment effects.

After a preliminary checkerboard assay a concentration of 16 µg/mL CaO_2_ NP was selected for studying the effect on 48 h-old biofilms. In combination with Tob at 64, 128 and 256 µg/mL a significantly enhanced antibacterial activity (approximately 2 log CFU/mL) for CaO_2_ NP was observed compared to treatment with Tob alone (Fig. 2b). Analogous experiments were conducted with CaCl_2_ at the same concentration as CaO_2_ NP i.e. 16 µg/mL and the same concentrations of Tob. It was found that CaCl_2_ also increased the antimicrobial effect of Tob, but always to a significantly lower intensity (approximately 1 log CFU/mL) than CaO_2_ NP.

For the older 72 h biofilm, it was necessary to increase the concentration of CaO_2_ NP to 32 µg/mL. It was observed that at 64 µg/mL Tob was no longer active regardless of the presence or absence of any Ca materials. However, at higher Tob concentrations (128 and 256 µg/mL) the enhancement of the antimicrobial activity by both Ca-materials was the same as for the younger biofilms, but leading to complete eradication only in presence of CaO_2_ NP. Again the enhancement by CaCl_2_ was less pronounced than by CaO_2_ NP. For a comparison between concentrations used in the in situ experiments, 96 µg/mL CaO_2_ NP alone and in combination to 128 µg/mL Tob were tested. In this case the bacterial viability was reduced by a 0.5 log CFU difference in combination treatment when compared with 32 µg/mL CaO_2_ NP and 128 µg/mL Tob (Supplementary Fig. 4).

To study the effect of oxygen delivery in absence of calcium, we also used O_2_-PFH liposomes, which were able to dissolve and hold oxygen due to the perfluorohexane (PFH) in its core, at the same concentration of 300 µg/mL as previously reported Hu et al. as an enhancer for antibiotic therapy^41^. Tob was only used at the highest concentration of 256 µg/mL, but otherwise we followed the same experimental protocol. It was found that O_2_-PFH liposomes also enhanced the activity of Tob, but the effect was less pronounced than for the Ca-materials, regardless of the approximately 10times higher concentration of the enhancer.

### Microscopical and morphological analysis of PAO1 biofilm

**Fig. 3:**
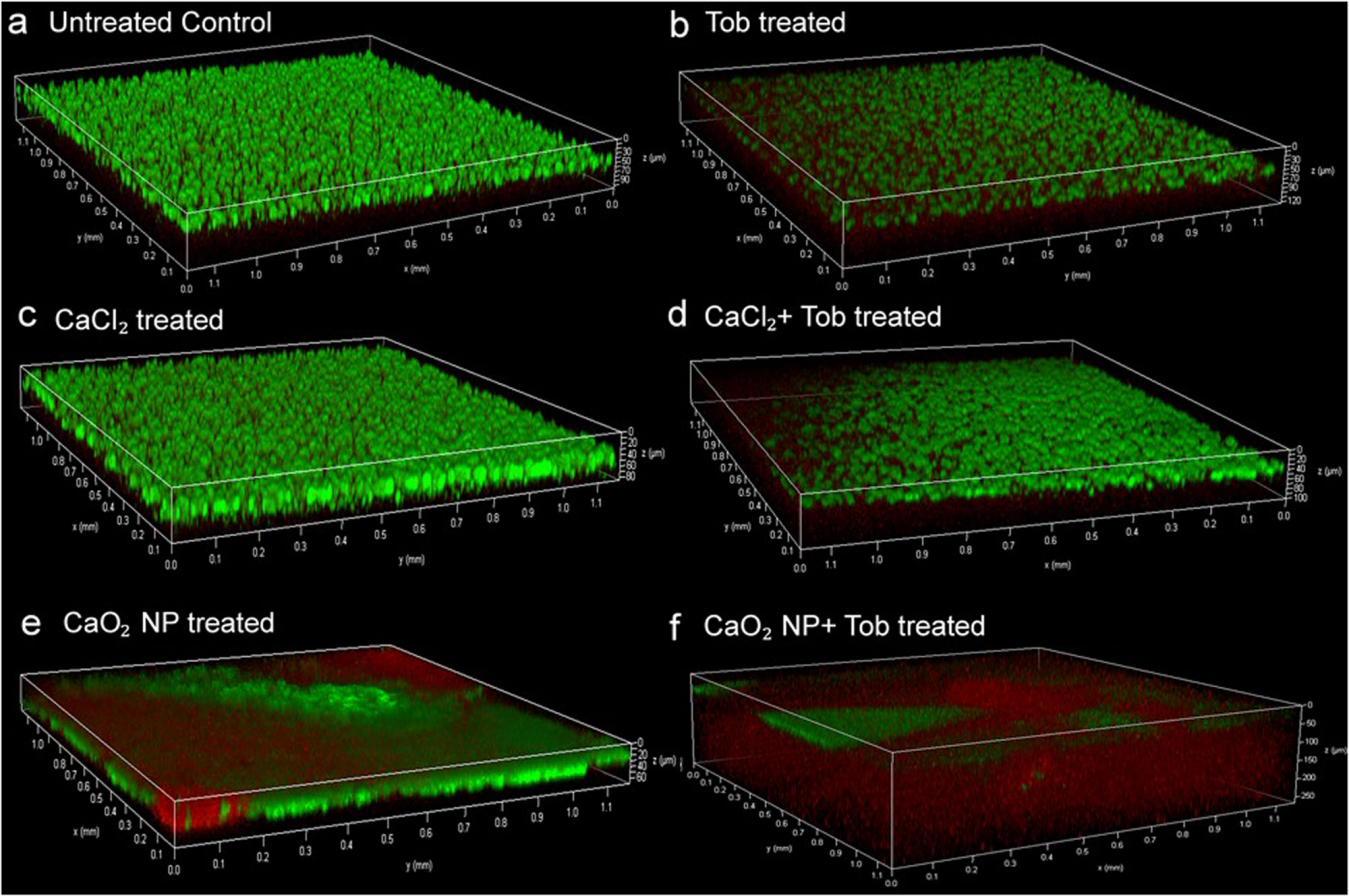
Confocal laser scanning microscopic (CLSM) analysis of 72 h old PAO1 biofilm, using Live/Dead® BacLight™ staining, live bacteria indicated by green fluorescence signal and dead bacteria indicated by red fluorescence signal. Overlay of green and red fluorescence in the represented images observed under 10X objective. (**a**) Untreated Control, (**b**) 256 µg/mL Tob treated, (**c**) 32 µg/mL CaCl_2_ treated (**d**) 32 µg/mL CaCl_2_ and 256 µg/mL Tob co-treated (**e**) 32 µg/mL CaO_2_ NP treated (**f**) 32 µg/mL CaO_2_ NP and 256 µg/mL Tob co-treated. The antibiotic potentiating effect of CaO_2_ NP for Tob is clearly visibile from the analyzed samples.

The 72 h PAO1 biofilms, treated with 32 µg/mL CaO_2_ NP or CaCl_2_ with 256 µg/mL Tob were further analyzed microscopically by the confocal laser scanning microscopy (CLSM). The Live/Dead® staining kit indicates, by using the green SYTO9 signal live bacteria, and red propidium iodide signal for bacteria with cell envelope leakage, respectively. The images in Fig. 3 represent the overlay for green and red fluorescence. We found that equivalent amounts of staining solutions for treated and untreated biofilm samples should not be used. The differences in biomass between the two samples otherwise affects the image quality, resulting in uneven distribution of green and red fluorescence in the biofilm. Thus, the method was modified considering the biomass in the wells, with a larger amount of staining solution for the untreated samples compared to treated samples with reduced biofilm. This adjustment caused the stain to evenly distribute and the fluorescence to be comparable between treatment groups.

We observed that the presence of green fluorescence, indicative for viable bacteria, was different between the central and peripheral parts of the well. In the middle of the well, the live bacteria were found in the upper and at the sides in the lower layer. Along the z-axis the onset of green fluorescence allowed to identify the air-interface of the biofilm in all samples. The thickness of biofilm observed was 50 µm for the untreated control (Fig. 3a) and 32 µg/mL CaCl_2_ (c) treated samples. Treatment by 256 µg/mL Tob (b) or 32 µg/mL CaO_2_ NP (e) alone reduced biofilm thickness to 30 µm. The combined treatment with 32 µg/mL CaCl_2_ and 256 µg/mL Tob (d) reduced biofilm thickness even further to 20 µm. However, for the combination of 32 µg/mL CaO_2_ NP with 256 µg/mL Tob (f) biofilm thickness was estimated to be >100 µm, but the red fluorescence indicated that the majority of bacteria was dead, with only some small green patches at the corners of the well.

Looking at the green fluorescence signal the staining for live bacteria was distributed evenly in the untreated and Tob treated samples. However, for the samples treated with CaCl_2_ the green fluorescence at the frontal y-z section appeared brighter, indicating less spaces between biofilm clusters. CaO_2_ NP treated samples showed a patchy distribution of staining and the biofilms were tightly packed with green fluorescence as compared to untreated samples. When 32 µg/mL CaO_2_ NP treated biofilms were observed from the top, the images showed holes in the biofilms and a patchy distribution of the staining which is also confirmed by the 2D images (Supplementary Fig. 2e). The red fluorescence signal observed in the samples treated with 32 µg/mL CaO_2_ NP with 256 µg/mL Tob was relatively stronger than in other treated samples (Fig. 3f and Supplementary Fig. 2f).

**Fig. 4:**
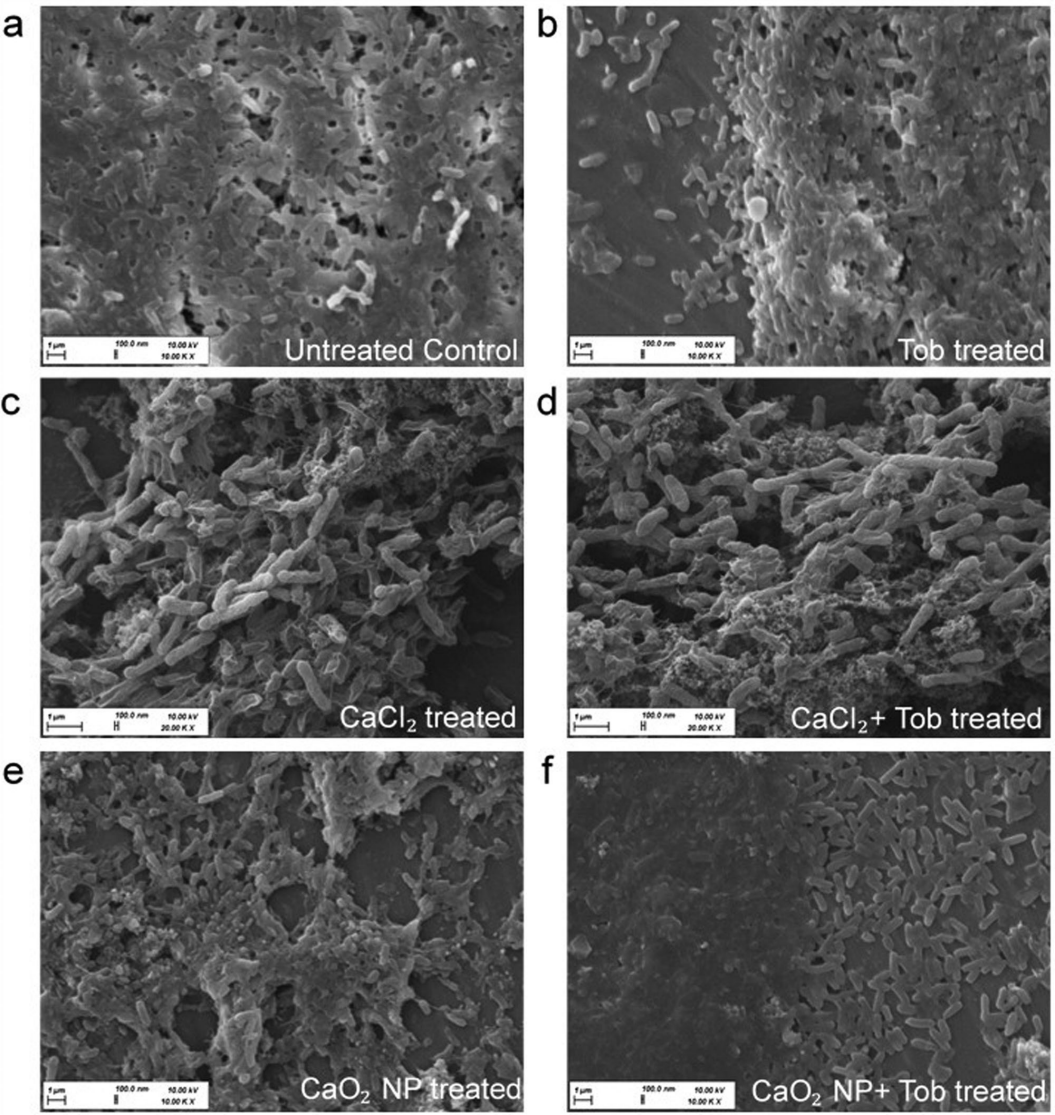
Morphological analysis of extracellular biofilm matrix for treated in vitro 72 h old PAO1 biofilm by SEM. (**a**) Untreated Control, (**b**) 256 µg/mL Tob treated, (**c**) 32 µg/mL CaCl_2_ treated (**d**) 32 µg/mL CaCl_2_ and 256 µg/mL Tob co-treated (**e**) 32 µg/mL CaO_2_ NP treated (**f**) 32 µg/mL CaO_2_ NP and 256 µg/mL Tob co-treated. The biomass for co-treated biofilms with CaO_2_ NP and Tob was significantly lower.

While the CLSM analysis indicated the intactness of the bacterial membranes for studying the bacterial viability, we also analyzed the extracellular biofilm matrix morphologically by SEM. It was observed from SEM micrographs, that the biofilm thickness was changed possibly due to the multiple washing steps during sample processing. Micrographs of the untreated control showed rod shaped bacteria completely covered in a matrix (Fig. 4a). When the biofilms were treated with 256 µg/mL Tob (Fig. 4b) the samples showed areas with biofilm eradication and also biofilm densification, it can also be seen that the bacteria were still covered with biofilm matrix. For the samples 32 µg/mL CaCl_2_ treatment (Fig. 4c, d) the biofilm had some salt deposits. The 32 µg/mL CaCl_2_ with 256 µg/mL Tob co-treated sample (Fig. 4d) showed a rough surface of remaining bacteria and shrinkage in membranes, indicating leakiness and compromised viability. Interestingly, the sample treated with 32 µg/mL CaO_2_ NP (Fig. 4e and Supplementary Fig. 1d), showed generation of pores in the biofilm matrix, and the bacteria were not visible in the image. The 32 µg/mL CaO_2_ NP with 256 µg/mL Tob co-treated sample (Fig. 4f) showed flat bacterial membranes outside the matrix, possibly due to dehydration of bacteria from the leaky membranes, and the biofilm matrix was significantly thin as compared to the 256 µg/mL Tob treated sample.

### Gene expression analysis of PAO1 in response to antimicrobial materials

Bacterial genetic expression responses were studied for the PAO1 biofilm model. For the gene expression analysis, concentrations of treatments were adjusted to retain enough biomass allowing the isolation of sufficient bacterial RNA for reproducible PCR analysis. 72 h old biofilm was treated with 32 µg/mL CaO_2_ NP or CaCl_2_ and Tob at 64 µg/mL in combination or alone. Genes of interest were selected as markers for changes due to treatment strategy in bacterial survival processes. Expression of genes such as rpoS, rpoD involved during the growth cycle of bacteria, encoding some housekeeping genes. pqsA, mvfR, rhlR are involved in the cell communication and biofilm virulence development. hcnA, phzA, nadB are associated with virulence related to micro-aerobic mode of respiration. mexG is annotated with a bacterial efflux pump. For this study, gyrA a genome replication housekeeping gene was selected as the reference gene. The isolated RNA was further converted to cDNA and qPCR studies were performed. The expression was calculated and displayed as fold expression change for each treatment group normalized to the untreated control group.

**Fig. 5:**
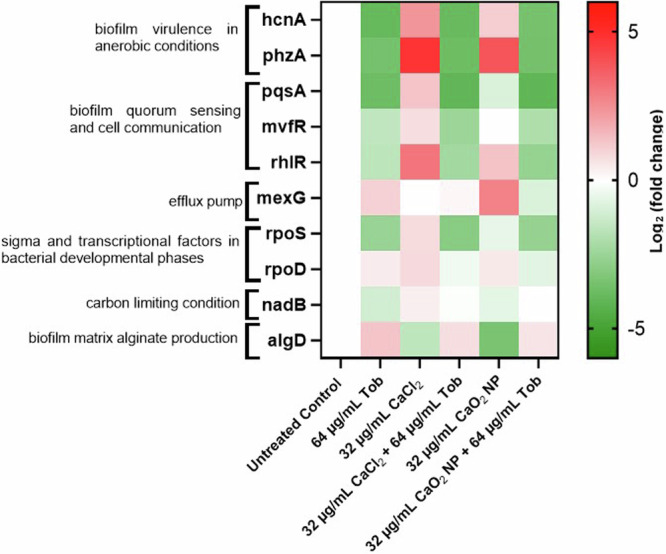
Gene expression analysis of in vitro 72 h old PAO1 biofilm in response to treatments. qPCR analysis performed for genetic expression of selected genes- hcnA, phzA, nadB, pqsA, mvfR, rhlR, mexG, rpoS, rpoD, algD. Tob treatment down-regulated gene expression as to the untreated control. CaCl_2_ treatment had a strong upregulation of biofilm quorum sensing, efflux mechanism and virulence factors, which was suppressed by the co-treatment with Tob. CaO_2_ NP had a similar but less pronounced effect as that from CaCl_2_, the combination of CaO_2_ NP and Tob down-regulated the gene expression.

Several genes were upregulated after administering calcium treatments (Fig. 5). The upregulation of biofilm virulence due to phzA, upon 32 µg/mL CaO_2_ NP and CaCl_2_ treatments respectively, by 3.8 and 4.7 log_2_ folds was observed. However, this was downregulated to −3 log_2_ folds when Tob was used in treatment alone or in combination to calcium materials. This expression analysis trend was followed by several other biofilm virulence representing genes like hcnA, pqsA, mvfR, rhlR, and rpoS. We noticed that in the combination of 32 µg/mL CaO_2_ NP and 64 µg/mL Tob the gene expression downregulated for hcnA, phzA, pqsA by nearly −3.5 to −4 log_2_ folds; whereas gene expression for rpoS, mvfR, and rhlR downregulated by −2.5 to −3 log_2_ folds. The expression analysis of bacterial defense represented by mexG and rpoD, it was observed that after treatment with 32 µg/mL CaO_2_ NP and CaCl_2_, and 64 µg/mL Tob the genetic expression was upregulated to 2.7, 7.2, 1 and 0.5, 0.8, 0.4 log_2_ folds, respectively. However, after a combination treatment with 32 µg/mL CaO_2_ NP/ CaCl_2_ and 64 µg/mL Tob the bacterial defense was downregulated to a nearly −0.8 log_2_ folds. Biofilm matrix production was affected due to the presence of Tob, in combination to calcium or used alone, the gene expression was upregulated to nearly 0.7 log_2_ folds. However, samples treated with CaO_2_ NP/ CaCl_2_ showed a downregulation in biofilm matrix production to −3.3 and −1.6 log_2_ folds, resp.

### In vitro control experiments on PAO1 biofilm

In an attempt to better understand the importance of ROS/oxygen and Ca^++^ for the additive antimicrobial effect, two controls were included in the design of experiments on 72 h PAO1 in vitro biofilms:

First, O_2_-PFH liposomes were used to compare the CaO_2_ NP mediated delivery of ROS to a reported liposomal delivery system for just O_2_. Parts of the results were already included in Fig. 2c for direct comparison. To differentiate the effect of PFH and O_2_-PFH in the liposomes, they were tested at both 36 and 300 µg/mL concentrations (Supplementary Fig. 1c). We observed that only biofilms treated with higher PFH-liposome concentration showed enhanced antibacterial activity (approximately by 1.5 log CFU/mL). The O_2_-PFH liposome at higher concentrations were 3 log CFU/mL more effective in decreasing bacterial growth as to when used at lower concentration.

**Fig. 6:**
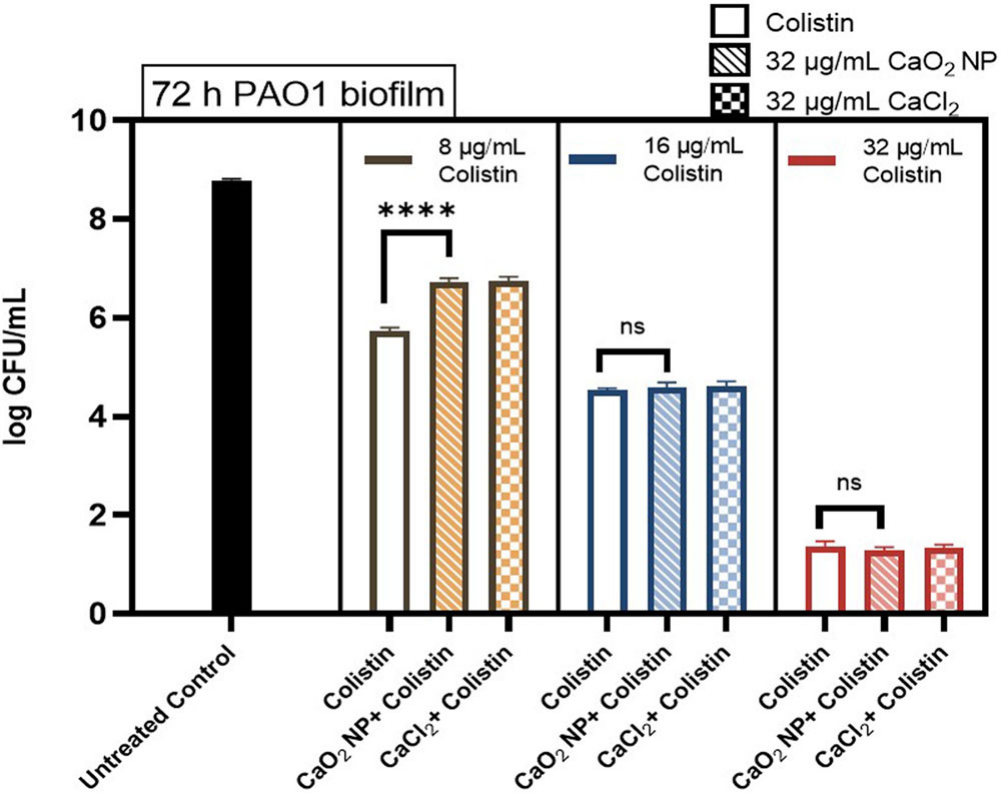
CFU assay for in vitro 72 h old PAO1 biofilm- treated with 32 µg/mL CaO_2_ NP/ CaCl_2_; with antibiotic colistin at 8, 16, and 32 µg/mL concentrations respectively. Nearly 1 log CFU/mL increased bacterial viability in combination treatment of 32 µg/mL CaO_2_ NP/ CaCl_2_ with 8 µg/mL colistin as compared to 8 µg/mL colistin alone. Experimental analysis performed using One-Way ANOVA with Tukey’s post hoc test. N*n = 3*3, ****p < 0.0001.

Secondly, we investigate colistin as an alternative antibiotic to Tob, in particular because it is reported to use the calcium channel for cellular entry^55^ and its antimicrobial activity might therefore be affected by Ca-materials^56^. 32 µg/mL CaO_2_ NP or CaCl_2_ were first added to the biofilm, 2 h later were followed by colistin at 8, 16 and 32 µg/mL and overnight incubation. It was observed from CFU viability assay (Fig. 6) that when colistin was used at a lower concentration of 8 µg/mL alone the bacterial survival was approximately 1 log CFU/mL lower than compared to the combination with Ca-materials. However, at higher colistin concentrations of 16 and 32 µg/mL the bactericidal effect observed was independent to the activity from CaO_2_ NP and CaCl_2_ (Fig. 6).

### Effect on in situ grown human oral biofilms

**Fig. 7:**
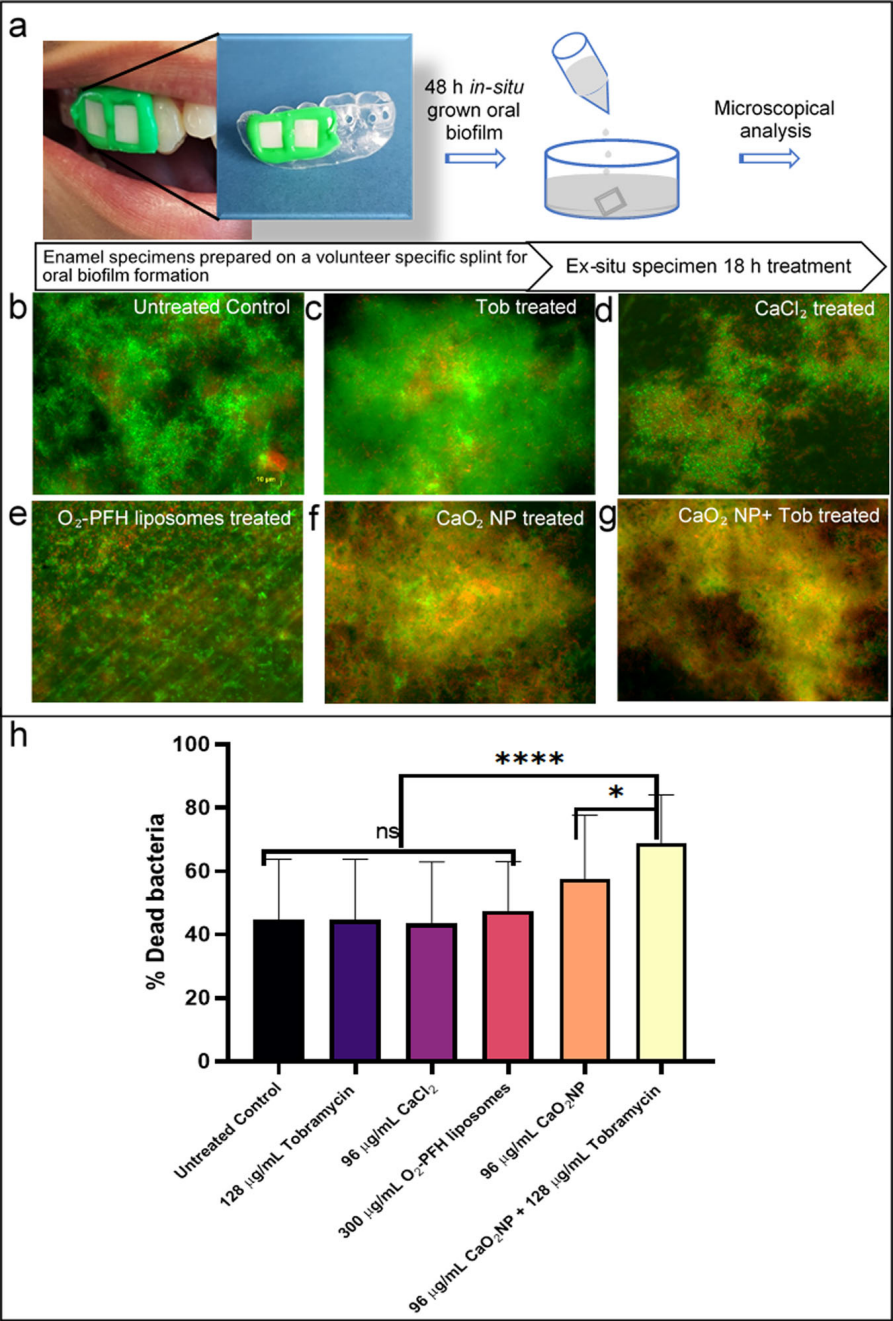
In situ grown oral biofilm treatment. (**a**) Schematic representation for treatment protocol. 48 h in situ grown oral biofilm- treated for 18 h ex situ, further analyzed using fluorescence microscopy, using Live/Dead® BacLight™ staining, live bacteria indicated by green fluorescence signal and dead bacteria indicated by red fluorescence signal. Overlay of green and red fluorescence in the represented images observed under 100X objective; Images for treated samples as follows. (**b**) Untreated Control, (**c**) 128 µg/mL Tob, (**d**) 96 µg/mL CaCl_2_, (**e**) 300 µg/mL O_2_-PFH liposomes, (**f**) 96 µg/mL CaO_2_ NP, (**g**) 96 µg/mL CaO_2_ NP + 128 µg/mL Tob co-treated. (**h**) Graph representing percentage of dead bacteria from treated specimens calculated using MATLAB software. Results showed approximately 13% enhanced bacterial elimination in CaO_2_ NP alone treated samples, whereas 23% enhanced bacterial death in CaO_2_ NP + Tob co-treated samples to that of the Untreated Control. Experimental analysis performed using One-Way ANOVA with Tukey’s post hoc test. *p < 0.05, ****p < 0.0001.

After observing promising effects of the CaO_2_ NP and Tob co-treatment in in vitro biofilms, we explored the same strategy for oral biofilm treatment. The in situ grown 48 h oral biofilms were treated ex situ with the testing solutions for 18 h. (Fig. 7a). Initially, to confirm the activity of Tob, it was tested at different concentrations of 1000, 500, 250 and 128 µg/mL. The bacterial viability was analyzed by fluorescence after live/dead staining. The antimicrobial effect of Tob was observed extensively till 250 µg/mL (Supplementary Fig. 3a–d). At 128 µg/mL the antimicrobial effect of Tob was minimal (Fig. 7c), thus we selected this sub-lethal concentration to observe the co-treatment activity with CaO_2_ NP. To identify the concentration of CaO_2_ NP required to show antimicrobial effect, we used 32, 96, 144 and 192 µg/mL (Supplementary Fig. 3e–h). 96 µg/mL CaO_2_ NP showed the required activity (Fig. 7f). Thus, further experiments were performed using 96 µg/mL CaO_2_ NP and 128 µg/mL Tob. The controls used in these experiments were CaCl_2_ at the same concentration to CaO_2_ NP (96 µg/mL) and 300 µg/mL O_2_-PFH liposomes. The percentage of dead bacteria were calculated based on total cells detected (living and dead) compared to dead cells, using the MATLAB image analysis software.

In in situ grown biofilms, a certain percentage of bacteria in the control sample was found to be dead. The mean values of percentage dead bacteria in untreated control, samples treated with 128 µg/mL Tob, 96 µg/mL CaCl_2_ and 300 µg/mL O_2_-PFH liposomes were around 45%. However, for the samples treated with CaO_2_ NP the percentage of dead bacteria was significantly increased to 57%, and for samples treated with CaO_2_ NP + Tob combination, it increased to 69% (Fig. 7h). Our results show an antimicrobial effect of CaO_2_ NP on native oral biofilms, which is enhanced when combined with Tob treatment.

## Discussion

The calcium peroxide nanoparticles prepared for this study had a spherical morphology, with a particle size of around 120 nm. However, as a dry powder CaO_2_ NP showed aggregation, possibly due to the attractive forces between them, but were easily dispersible in ethanol^55,56^. The physical characteristics of CaO_2_ NP considering the size, morphology and ability to release oxygen were comparable to other studies conducted on such particles^12,35^. When oxygen generation capacity of CaO_2_ NP was compared to the control with oxygen releasing O_2_-PFH liposomes, it was observed that later increased the oxygen concentration faster than CaO_2_ NP. When considering this phenomenon in treatment of hypoxic biofilms, it could be speculated that a gradual release of oxygen from CaO_2_ NP could be more beneficial. In addition, CaO_2_ NP can also deliver H_2_O_2_ which is a ROS, as an intermediate before oxygen release^38^. This ROS intermediate can also have an effect on biofilm; as it is used in different studies in controlling biofilm growth^57,58^. The effect of calcium peroxide, in the works of Shen et al. was studied on bacteria such as *Escherichia coli*, *Clostridium tetani*, and *Fusobacterium nucleatum* to study the enhancement of bacteriostatic effect of smaller sized particles; and of Qi et al. on the effect of wound healing with calcium peroxide embedded in polycaprolactone and gelatin fiber which also were able to eliminate *E. coli* growth. However, these studies were limited to planktonic bacteria treatment^14,15^. To study the effect of CaO_2_ NP on in vitro biofilms of pathogenic *P. aeruginosa*, we used the lab-strain PAO1. For treatment of biofilms, concentrations of 16 and 32 µg/mL were selected considering the biocompatibility of CaO_2_ NP. When 48 h and 72 h PAO1 biofilms were treated, a dose dependent additive effect was observed from CaO_2_ NP with Tob. When the CaO_2_ NP come in contact with water they not only released oxygen but also a calcium hydroxide byproduct. To confirm that the calcium also had effect on cells^59^; calcium chloride was added as a control to these experiments. Similar concentrations of calcium chloride were used to that of CaO_2_ NP to treat the PAO1 biofilms. Our results showed that calcium decreases bacterial viability only in combination with Tob (Fig. 2b, c). They do obviously so in the dual capacity of releasing both calcium and oxygen. The Tob concentration required for complete eradication of PAO1 biofilm was significantly reduced when combined with CaO_2_ NP (Fig. 2). When the biofilms were grown for different times, i.e. 48 and 72 h, the dose required for eradication in the assay increased^60^. These results indicate that the antimicrobial resistance requiring higher doses of antibiotics for treatment of biofilms can be reduced by using such strategy. As the results indicated that the CaO_2_ NP release both oxygen and calcium, additional experiments based on separate delivery of oxygen and calcium, resp., were performed. Calcium free O_2_-PFH liposomes were tested on the 72 h PAO1 in vitro model. In the work of Hu et al. O_2_-PFH liposomes had been tested before in combination with several antibiotics on 24 h *P. aeruginosa* biofilms, which was explained by relieving hypoxia in biofilms by oxygen generation^41^. In our studies the combination of O_2_-PFH liposomes and Tob had a significant effect on bacterial viability, but the combination of Tob with CaCl_2_ was more effective, while the combination with CaO_2_ NP even led to a complete biofilm eradication. We also tested colistin in combination with the CaO_2_ NP, as it has been speculated that the bactericidal effect of colistin may involve the calcium channels in gram negative bacteria^55^. However, we found the antibacterial effect of colistin at low concentration (8 µg/mL) reduced in combination with CaO_2_ NP, and no additional effect when colistin was used at higher concentrations.

Considering the maturity of biofilms, 72 h PAO1 biofilms were analyzed further by different techniques. The treated in vitro biofilms were analyzed microscopically by CLSM and SEM to investigate the bacterial viability and the extracellular matrix structure. As the biofilms were grown in M63 medium the addition of calcium during treatment, results in a metabolic shift during bacterial survival which releases additional biofilm extracellular polymeric substances^61^. Our results suggest that holes and pores were formed in the biofilm treated with CaO_2_ NP, possibly due to the intermediate H_2_O_2_ effect. The morbidity of bacteria were observed morphologically with SEM analysis, and CLSM analysis also confirmed with enhanced red fluorescence that the samples co-treated with CaO_2_ NP and Tob had higher bacterial elimination.

Biofilms were also analyzed for gene expression in response to the different treatments. A sub-lethal concentration of Tob (64 µg/mL) was chosen to retain sufficient bacteria for analyzable quantities of RNA. It was observed that the biofilm virulence related genes hcnA, phzA, pqsA, mvfR, rhlR, rpoS in response to the treatment with 32 µg/mL CaO_2_ NP or CaCl_2_ were upregulated, indicating that the calcium materials affected the metabolism in bacteria. However, this effect was suppressed in combination with 64 µg/mL Tob, resulting in decreased survival of bacteria. The expression of biofilm alginate production gene algD, interestingly downregulated in response to CaO_2_ NP or CaCl_2_ treated samples. The results suggest that the bacterial survival and metabolism was switched from the biofilm mode. However, when the same expression analysis was observed in the presence of 64 µg/mL Tob, it was slightly upregulated. The expression analysis involving bacterial defense by genes like mexG and rpoD, observations indicated that for samples treated with either CaO_2_ NP or CaCl_2_ and Tob they were obviously upregulated. Though, in combination treatments the defense strategies were downregulated. These results indicated that CaO_2_ NP affect bacterial virulence and immune response, which may explain the increased susceptibility of PAO1 biofilms to Tob. As the combination of CaO_2_ NP and Tob potentiated the effect of bacterial killing, it was also observed by the downregulation of gene expression several virulence and immune response related genes.

For the treatment of native oral biofilms, the concentration of CaO_2_ NP were chosen on the safety recommendations for calcium peroxide provided by the scientific committee on consumer products of the European Commission. A dose of 0.5% of pea-sized, 0.25 g of toothpaste was considered safe^22,62^. Therefore, 96 µg/mL CaO_2_ NP concentration was used in the treatment of in situ biofilms. In the work of Müller-Heupt et al., mono-species 24 h old biofilm of *P. gingivalis* and *Streptococcus oralis*, were studied in vitro for antibacterial effect of calcium peroxide. According to their results, 500 µg/mL calcium peroxide was required to eliminate *P. gingivalis* biofilm, whereas the *S. oralis* biofilm was not eradicated^17^. However, in our study on in situ grown multi-species biofilm concentrations of 96 µg/mL CaO_2_ NP the percentage of dead bacteria increased by 13%, whereas for the combination treatment of 96 µg/mL CaO_2_ NP and 128 µg/mL Tob it increased by 24%. While CaO_2_ NP obviously have some intrinsic antimicrobial activity, the combination with Tob can additionally enhance the antimicrobial effect.

In summary, our work demonstrates that CaO_2_ NP when used in combination with the antibiotic Tob, leads to an increased antibacterial activity. The original hypothesis to prepare CaO_2_ NP was to generate oxygen in the biofilms, making them more susceptible to antibiotic treatment. However, we found that the CaO_2_ NP were not only able to produce oxygen, but also the contained calcium enhanced bacterial killing. In vitro analysis of CaO_2_ NP indicated that they potentiate the antimicrobial activity of Tob by acting in this dual capacity. CaO_2_ NP might facilitate the penetration of Tob into the biofilm matrix and sensitize the bacteria within a biofilm towards antibiotic treatment. Gene expression analysis suggested that although calcium compounds alone increased the virulence of bacteria, the combination of Tob decreased it. In situ oral biofilm treatment not only indicated that the combination treatment of CaO_2_ NP and Tob was more beneficial in biofilm eradication, but also confirmed the potency of CaO_2_ NP as an antimicrobial agent. CaO_2_ NP used for teeth whitening could also show potential benefits in controlling oral microbial dysbiosis related infections.

### Reporting summary

Further information on research design is available in the Nature Research Reporting Summary linked to this article.

## Supplementary information

Reporting summary

Antimicrobial and antibiotic-potentiating effect of calcium peroxide nanoparticles on oral bacterial biofilms

## Data Availability

The datasets used and analyzed in the current study will be available from the corresponding author on reasonable request.
