# Supplementary material for: Antimicrobial and antibiotic-potentiating effect of calcium peroxide nanoparticles on oral bacterial biofilms
Source: NPJ Biofilms Microbiomes. 2024 Oct 15;10:106. doi: 10.1038/s41522-024-00569-7 (PMC11480382; doi:10.1038/s41522-024-00569-7)
Supplement: Supplementary file 2 — Antimicrobial and antibiotic-potentiating effect of calcium peroxide nanoparticles on oral bacterial biofilms [file 41522_2024_569_MOESM2_ESM.pdf]

Antimicrobial and antibiotic-potentiating effect of calcium peroxide nanoparticles on oral bacterial biofilms

Neha Bankar, Lorenz Latta, Brigitta Loretz, Bashar Reda, Johanna Dudek, Hendrik Hähl, Matthias Hannig, Claus-Michael Lehr[*]

* Corresponding author Email, claus-michael.lehr@helmholtz-hips.de

Supplementary Figures

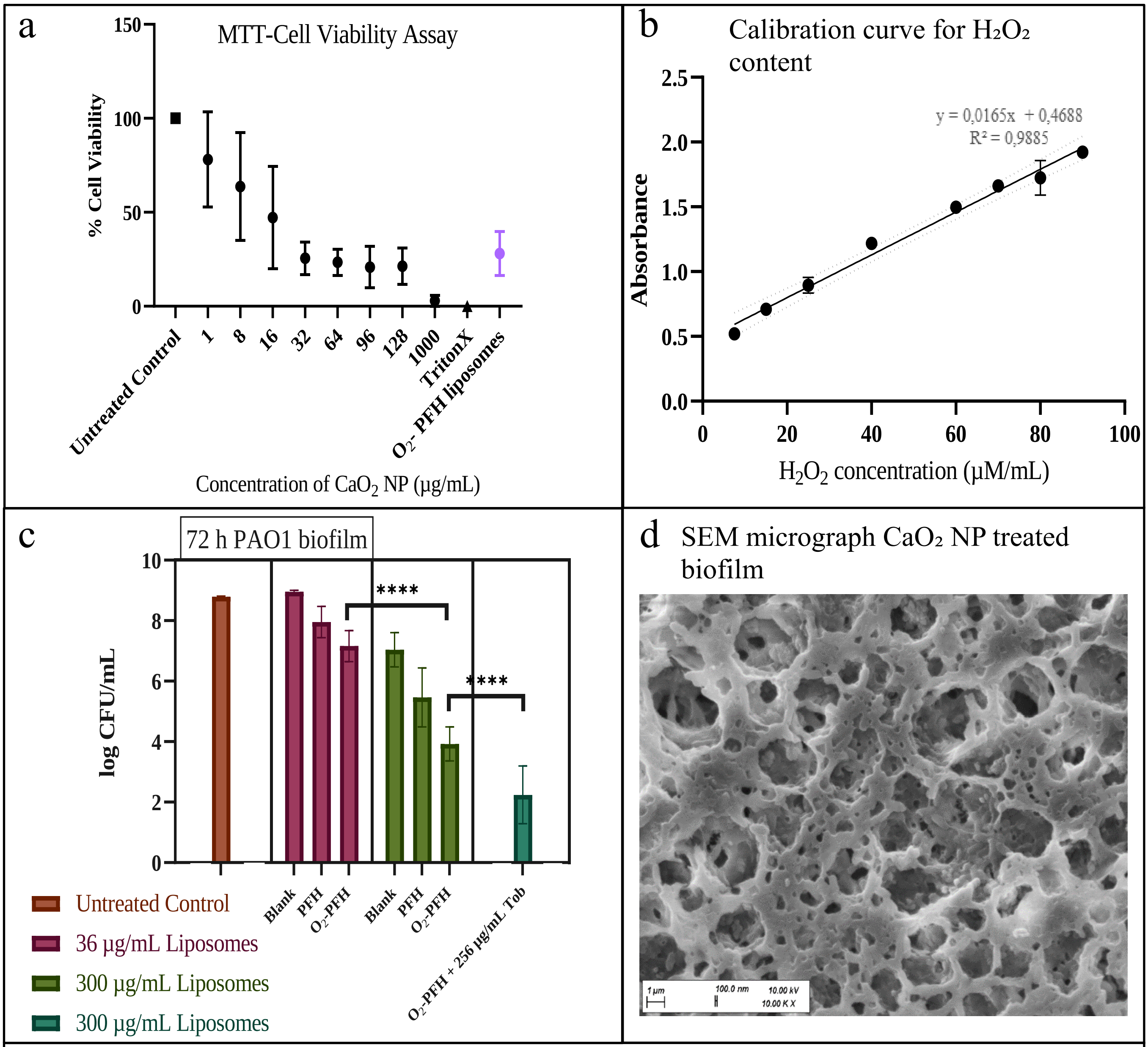

*Supplementary Figure 1 (a) MTT- cell viability assay, gingival fibroblast cells cultured for 48 h were exposed to CaO_2 NP for 4 h, treated with MTT reagent, and the readout was made for cell viability. 50 % lethal concentration of CaO_2 NP was at 16 µg/mL (N*n=3*3). (b) Calibration curve for H_2O_2 content by Pierce™ quantitative peroxide assay kit. 50 µg/mL CaO_2 NP (N*4) were able to generate 49 ± 20 µM/mL H_2O_2. (c) CFU assay for in-vitro 72 h old PAO1 biofilm treatment- with 36 and 300 µg/mL blank, PFH, O_2-PFH liposomes, O_2-PFH liposomes+ 256 µg/mL Tob. Decrease in bacterial viability at higher concentrations, 2.24 log CFU/mL at O_2-PFH liposomes with 256 µg/mL Tob combination. (d) Morphological analysis of extracellular biofilm matrix for 32 µg/mL CaO_2 NP treated in-vitro 72 h old PAO1 biofilm by SEM, showing pore formation in the matrix.*

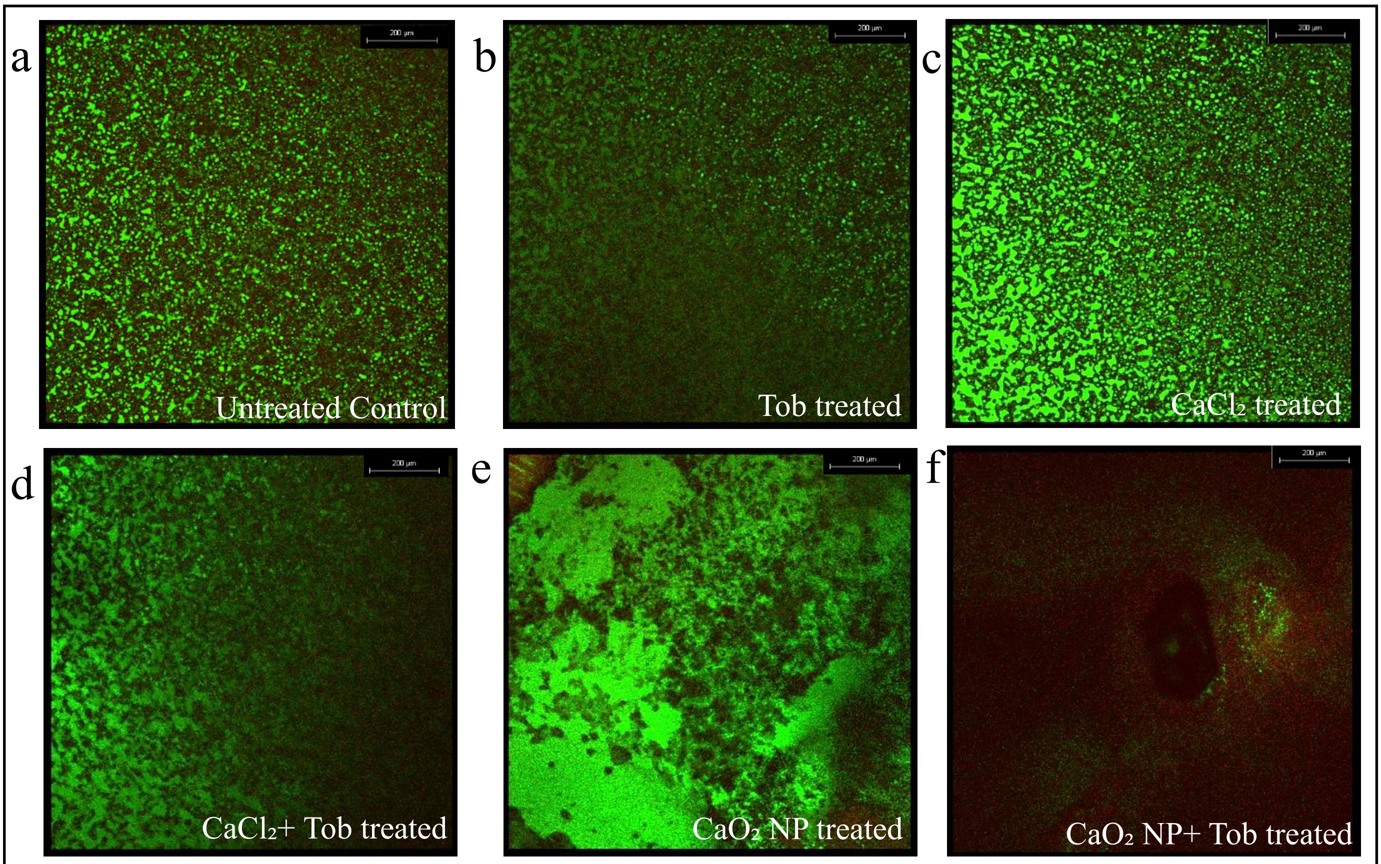

Supplementary Figure 2: Confocal Laser Scanning Microscopic (CLSM) analysis of in-vitro 72 h PAO1 biofilm (2 dimensional images), using Live/Dead® BacLight™ staining, live bacteria indicated by green fluorescence signal and dead bacteria indicated by red fluorescence signal. Overlay of green and red fluorescence in the represented images observed under 10X objective. (a) Untreated Control, (b) 256 µg/mL Tob treated, (c) 32 µg/mL $CaCl_2$ treated (d) 32 µg/mL $CaCl_2$ and 256 µg/mL Tob co-treated (e) 32 µg/mL CaO_2 NP treated (f) 32 µg/mL CaO_2 NP and 256 µg/mL Tob co-treated. The CaO_2 NP treated biofilm showed generation of holes in the biofilm.

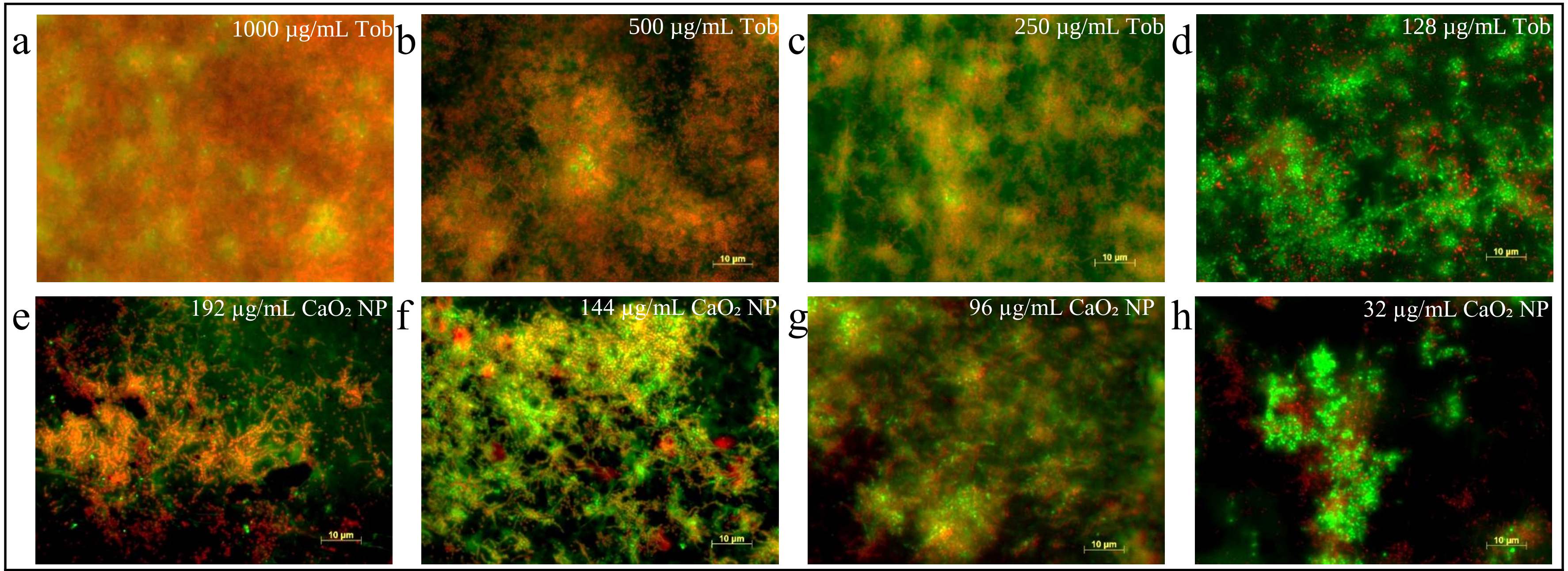

Supplementary Figure 3: 48 h in-situ grown oral biofilm treated overnight ex-situ further analyzed by- fluorescence microscopical analysis, using Live/Dead® BacLight™ staining, live bacteria indicated by green fluorescence signal and dead bacteria indicated by red fluorescence signal. Overlay of green and red fluorescence in the represented images observed under 100X objective. Images for treated samples as follows, (a) 1000 µg/mL Tob, (b) 500 µg/mL Tob (c) 250 µg/mL Tob, (d) 128 µg/mL Tob (e) 192 µg/mL CaO_2 NP (f) 144 µg/mL CaO_2 NP (g) 96 µg/mL CaO_2 NP (h) 32 µg/mL CaO_2 NP. 96 µg/mL CaO_2 NP showed potential antimicrobial activity; while 128 µg/mL Tob concentration was selected as a sub-lethal dose to analyze the co-treatment efficacy.

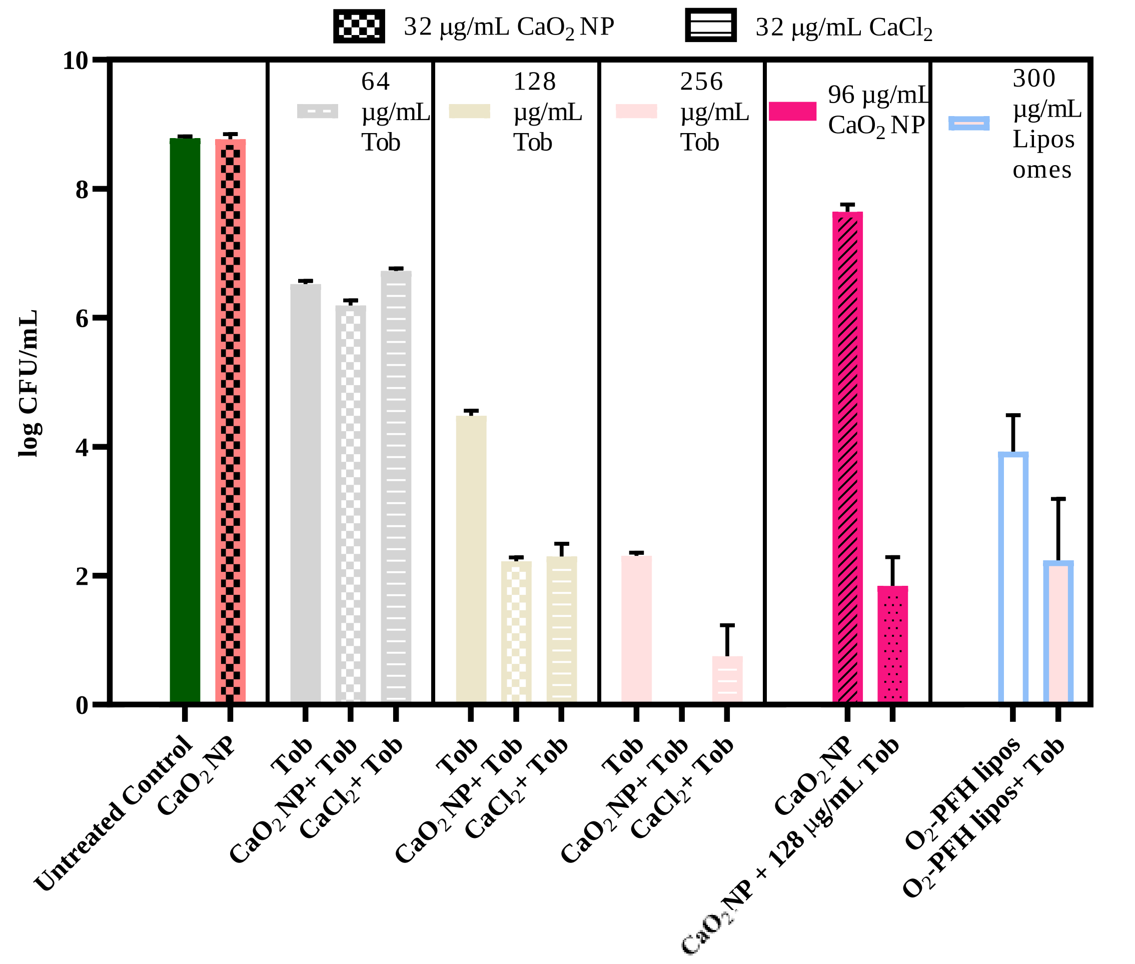

Supplementary Figure 4: CFU assay for in-vitro 72h old PAO1 biofilm- Comparison between 32 and 96 µg/mL CaO_2 NP alone and with antibiotic Tob at concentration of 128 µg/mL respectively. Bacterial viability was decreased by 0.5 log CFU/mL more in combination of 96 µg/mL CaO_2 NP and 128 µg/mL Tob when compared with 32 µg/mL CaO_2 NP and 128 µg/mL Tob.

Supplementary Table

Supplementary Table 1. Primer sets for qPCR analysis

Gene	Primer sequences
**gyrA Fw**	TGTGCTTTATGCCATGAGCGA
**gyrA Rev**	TCCACCGAACCGAAGTTGC
**hcnA Fw**	GGGCAGACATGACCATCCAC
**hcnA Rev**	ACGGCATTGAGCACGTTGAG
**phzA1 Fw**	GGCAACTGGACCACGGAAAG
**phzA1 Rev**	TGGATGTAGTGGTTCTCGCA
**pqsA Fw**	AGGCGGTTCTGGTTCCTACC
**pqsA Rev**	GCAGGATCTGGTTGTCGTCC
**rpoS Fw**	TCACCCGAAGAAATCGCCAA
**rpoS Rev**	AAGTCACCCGTTCGTTCAGG
**mexG Fw**	CTGTTCGACTATCAGGCCAGC
**mexG Rev**	CTCCAGAAGGTGTGGACGATG
**mvfR Fw**	TCGCCTGATCCCTTACATGC
**mvfR Rev**	TTGATCGTCGCCAGGCTATC
**RhlR Fw**	GGAGGCTTTTTGCTGTGGTG
**RhlR Fw**	ACTTCCTTTTCCAGGACGGC
**rpoD Fw**	GACTACAATCGCATCGTCGC
**rpoD Rev**	AATCGTCCTTCAGGTTGACCG
**nadB Fw**	CTACCTGGACATCAGCCAC
**nadB Rev**	GGTAATGTCGATGCCGAAGT
**algD Fw**	TGTCGCGCTACTACATGCGTC
**algD Rev**	GTGTCGTGGCTGGTGATGAGA

Primer sequence 5'-->3' in forward (Fw) or Reverse (Rev) manner
